# Oxylipin Profiling of Alzheimer’s Disease in Nondiabetic and Type 2 Diabetic Elderly

**DOI:** 10.3390/metabo9090177

**Published:** 2019-09-05

**Authors:** Jill K. Morris, Brian D. Piccolo, Casey S. John, Zachary D. Green, John P. Thyfault, Sean H. Adams

**Affiliations:** 1 Department of Neurology, University of Kansas Alzheimer’s Disease Center, Kansas City, KS 66205, USA; 2 University of Kansas Alzheimer’s Disease Center, Fairway, KS 66205, USA; 3 Arkansas Children’s Nutrition Center, Little Rock, AR 72205, USA (B.D.P.) (S.H.A.); 4 Department of Pediatrics, University of Arkansas for Medical Sciences, Little Rock, AR 72205, USA; 5 Department of Molecular and Integrative Physiology, University of Kansas, Kansas City, KS 66045, USA; 6 Kansas City VA Medical Center, Kansas City, MO 64128, USA

**Keywords:** oxylipin, alzheimer’s disease, type 2 diabetes, dihetre

## Abstract

Oxygenated lipids, called “oxylipins,” serve a variety of important signaling roles within the cell. Oxylipins have been linked to inflammation and vascular function, and blood patterns have been shown to differ in type 2 diabetes (T2D). Because these factors (inflammation, vascular function, diabetes) are also associated with Alzheimer’s disease (AD) risk, we set out to characterize the serum oxylipin profile in elderly and AD subjects to understand if there are shared patterns between AD and T2D. We obtained serum from 126 well-characterized, overnight-fasted elderly individuals who underwent a stringent cognitive evaluation and were determined to be cognitively healthy or AD. Because the oxylipin profile may also be influenced by T2D, we assessed nondiabetic and T2D subjects separately. Within nondiabetic individuals, cognitively healthy subjects had higher levels of the nitrolipid 10-nitrooleate (16.8% higher) compared to AD subjects. AD subjects had higher levels of all four dihydroxyeicosatrienoic acid (DiHETrE) species: 14,15-DiHETrE (18% higher), 11,12 DiHETrE (18% higher), 8,9-DiHETrE (23% higher), and 5,6-DiHETrE (15% higher). Within T2D participants, we observed elevations in 14,15-dihydroxyeicosa-5,8,11-trienoic acid (14,15-DiHETE; 66% higher), 17,18-dihydroxyeicosa-5,8,11,14-tetraenoic acid (17,18-DiHETE; 29% higher) and 17-hydroxy-4,7,10,13,15,19-docosahexaenoic acid (17-HDoHE; 105% higher) and summed fatty acid diols (85% higher) in subjects with AD compared to cognitively healthy elderly, with no differences in the DiHETrE species between groups. Although these effects were no longer significant following stringent adjustment for multiple comparisons, the consistent effects on groups of molecules with similar physiological roles, as well as clear differences in the AD-related profiles within nondiabetic and T2D individuals, warrant further research into these molecules in the context of AD.

## 1. Introduction

Oxygenated lipids (oxylipins) are a group of molecules derived from polyunsaturated fatty acids (PUFAs) that can mediate molecular signaling, including inflammatory pathways [1]. For example, eicosanoids are a well-known subset of oxylipins derived from arachidonic acid and other 20 carbon length PUFAs that serve as secondary messenger molecules, and are involved in processes of inflammation, oxidative stress, and vascular regulation [2,3,4]. Dysregulation of these factors has been implicated in both Alzheimer’s disease (AD) and type 2 diabetes (T2D) [5,6,7,8]. This would suggest that some T2D-associated oxylipin patterns might be shared with the AD condition (even without concurrent T2D). Previously, studies of blood oxylipin shifts in non-elderly adult women revealed that 18 carbon epoxides and ketones of PUFAs were elevated >80% and correlated with non-esterified fatty acids concentrations in T2D subjects [9].

The synthesis of some oxylipins is highly dynamic and may be affected by cell stress levels. This is relevant to AD because markers of oxidative stress, including lipid peroxidation, DNA, and protein oxidation levels are increased early in the disease processes [10,11,12]. At the same time, it has been shown that activation of a gene that is protective against oxidative stress declines with aging and is lost early in AD [13]. This could have marked effects on the oxidation of lipids, but it is unclear how specific oxylipin levels are affected in individuals with AD who are nondiabetic, and whether or not this profile is affected in AD subjects who also have T2D. A better understanding of these relationships could help identify novel AD biomarkers and help explain underlying processes that contribute to disease onset and progression. For this study, we leveraged a targeted assessment of oxylipins to evaluate changes in serum oxylipins between cognitively healthy elderly and AD subjects. We analyzed two groups: individuals who were nondiabetic or who had T2D. Our primary goal was to characterize the degree to which fatty acid metabolism and oxidation state are affected in AD subjects and whether these changes occur similarly in subjects with and without T2D. These experiments complement and extend our previously-published studies that compared the circulating “global metabolome” in the same cohort of subjects described here [14].

## 2. Results

We detected 46 oxylipin metabolites in the serum of 126 subjects. Demographic characteristics of this cohort have been published previously [14], and the groups did not differ by age, sex, or education. Within nondiabetic subjects, individuals with AD had higher serum concentrations of 14,15-DiHETrE, 11,12-DiHETrE, and 5,6-DiHETrE, and 8,9-DiHETrE, while concentrations of 10-nitrooleate were lower; however, these differences were not sustained after correcting for multiple comparisons (Table 1). Within T2D individuals, 14,15-DiHETE, 17,18-DiHETE, 17-HDoHE, and summed fatty acid diols were found to be significantly higher in AD subjects compared to cognitively healthy subjects (Table 2). Again, these findings were no longer considered statistically significant after adjusting for multiple comparisons. Notable roles for analytes identified within each group of subjects are summarized in (Table 3). No overall differences between nondiabetic and diabetic subjects were observed when these groups as a whole were compared directly, without considering AD status.

**Figure 1:**
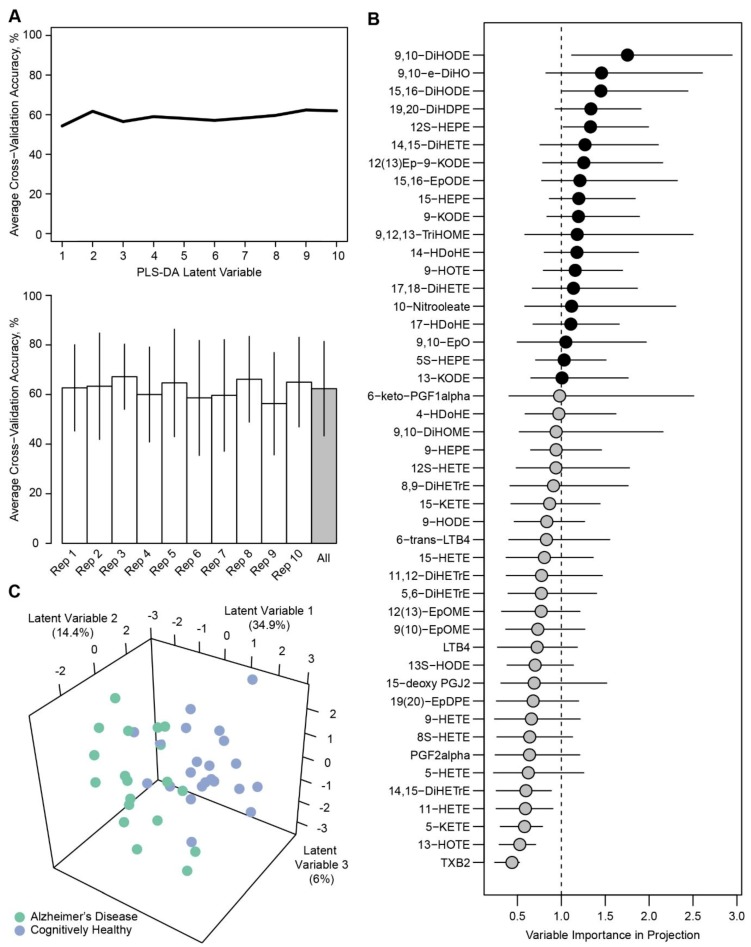
Modeling of Alzheimer’s disease (AD) status within type 2 diabetes (T2D) participants. (**A**) Cross-validation accuracy was 62.4% on average in cross-validation assessment. (**B**) Twenty-one metabolites had VIP scores >1, with three metabolites having bootstrapped VIP confidence intervals >1. (**C**) PLS-DA modeling with metabolites that had VIP >1 had higher cross-validation accuracy, with separation of PLS-DA scores apparent with first three latent variables.

Since the assessment of individual metabolites by univariate statistics did not reveal widespread differences (especially after FDR correction), we used a multivariate approach to determine whether global differences in the oxylipin metabolite signature could be elucidated. Using partial least squares discriminant analysis (PLS-DA) with all identified metabolites, modeling of AD status *within* T2D participants resulted in an average of 62.4% accuracy (nine latent variables) in cross-validation assessment (Figure 1A). A total of 21 metabolites had variable importance in projection (VIP) scores >1, with 9,10-DiHODE, 15,16-DiHODE, and 12S-HEPE having bootstrapped VIP confidence intervals >1 (Figure 1B). 14,15-DiHETE and 17,18-DiHETE, previously noted as statistically significant in univariate assessments, also had VIP calculations >1. PLS-DA modeling with metabolites that had a VIP >1 resulted in a slightly greater cross-validation accuracy (69.7% with five latent variables), suggesting a modest improvement in model performance when isolating potential discriminant metabolites. A slight separation of individual PLS-DA scores was observed across three latent variables in the reduced model (Figure 1C).

**Figure 2:**
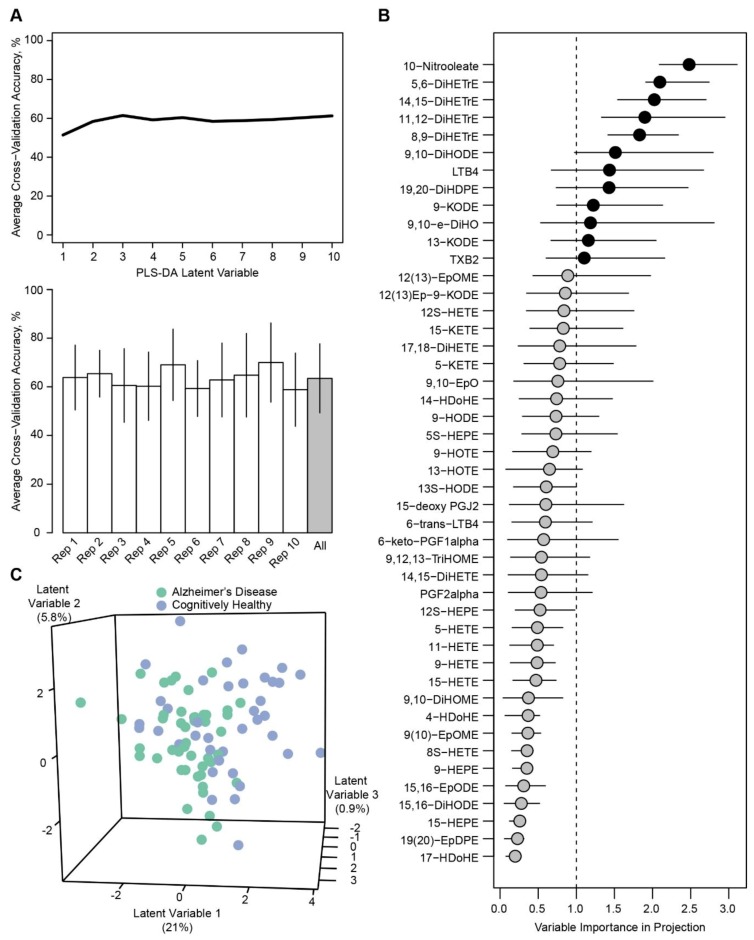
Modeling of AD status within subjects without T2D. (**A**) Cross-validation accuracy with 61.5% on average. (**B**) VIP assessment showed 12 oxylipins with VIP >1, with five metabolites having bootstrapped VIP confidence intervals >1. (**C**) PLS-DA modeling of metabolites with VIP >1 improved cross-validation accuracy, but visual separation of individual PLS-DA scores was not readily apparent with the first three latent variables.

PLS-DA assessment of AD status in subjects without T2D resulted in an average of 61.5% cross-validation accuracy with three latent variables (Figure 2A). VIP assessment showed 12 oxylipins with VIP >1 (Figure 2B). In concordance with the univariate results, 10-nitrooleate, 5,6-DiHETrE, 14,15-DiHETrE, 11,12-DiHETrE, and 8,19-DiHETrE had bootstrapped 95% confidence intervals >1. PLS-DA modeling of oxylipins with VIP >1 also improved average cross-validation predictions to 69.0% with one latent variable; however, visual separation of individual PLS-DA scores was not readily apparent with the first three latent variables (Figure 2C).

## 3. Discussion

Although the etiology of AD is complex, there is broad evidence for increased oxidative stress. Previous reports have found alterations in blood and brain PUFAs in AD compared to cognitively healthy groups, and individuals with AD have lower levels of various plasma phosphatidylcholine species compared to cognitively healthy elderly [28]. AD subjects also have lower levels of several unsaturated fatty acids, including arachidonic acid, in the brain [29,30]. Finally, individuals with AD have lower soluble levels of the receptor for advanced glycation end products (RAGE) regardless of T2D diagnosis [31], which is postulated to reflect deficits in inflammatory control [32]. However, much less is known about AD-related effects on the oxylipin profile, or how T2D affects the oxylipin profile within AD subjects. This is important given T2D’s prevalence in elderly populations and its known impact on AD risk [33,34]. We only found a few oxylipins that were altered by AD. Interestingly, the majority of the altered were fatty acid diol species, formed by the hydrolysis of epoxy fatty acids by soluble epoxide hydrolase [35]. While these fatty acid diols were also identified in our PLS-DA modeling, the poor predictive performance of these models suggested that global alterations of oxylipins do not occur with AD. Still, the increase of several serum fatty acid diols found in subjects with AD, regardless of T2D status, suggests that epoxy fatty acid metabolism is altered in the disease.

The cytochrome P-450 (CYP) pathway, otherwise known as the epoxygenase pathway, is a branch of the arachidonic acid cascade, a pathway involved in inflammation [24]. A growing body of evidence suggests that CYP-related oxylipins may affect cardiovascular function, demonstrating both anti-hypertensive and pro-hypertensive effects depending upon the model studied and specific oxylipin [36]. CYP metabolites can also serve as secondary messengers for a variety of growth factors that regulate the cardiovascular system [37], especially in at-risk populations [22]. CYP enzymes, in general, have been shown to increase the release of pituitary hormones, such as somatostatin, vasopressin, and growth hormones through mechanisms related to calcium release and entry [36].

In nondiabetic individuals, all four serum epoxygenase (CYP)-derived dihydroxyeicosatrienoic acids (DiHETrEs) that we tested were higher by 15% to 23% in AD compared to cognitively healthy elderly. DiHETrEs are generally accepted to be “non-active” (or substantially less active) metabolite products of soluble epoxide hydrolase acting on epoxyeicosatreinoic acids (EETs) [38]. Though little is known about their function, arachidonic acid-related oxylipins, such as DiHETrEs, increase with fish oil supplementation [39]. This may be of note given clinical trials showing the benefit of fish oil supplements in AD [40], but additional studies are needed to establish mechanistic relationships. In addition, we identified one nitrolipid, 10-nitrooleate, that was lower in the nondiabetic AD group. Nitrooleate has been shown to promote protective vascular benefits by acting as a PPARγ ligand, as well as through increasing the availability of nitric oxide [15]. There is evidence that vascular dysfunction can increase the risk for AD and potentially contribute to cognitive decline [41]. Prior studies have associated the DiHETrE oxylipins with vascular function and vascular events (Table 3) and suggest that vascular function related changes in oxylipin metabolites occur in AD independent of T2D. Although the DiHETrE metabolites did not withstand stringent multiple comparisons statistical correction, the effects of these species on similar vascular-related physiological processes warrant further investigation.

Within T2D subjects, 14,15-DiHETE, 17,18-DiHETE, 17-HDoHE, and total fatty acid alcohols were significantly increased by 29% to 105% with AD (albeit not after multiple comparisons correction). Interestingly, 17,18-DiHETE is also increased in the brains of APP/tau mice, which exhibit amyloid pathology [42]. Regulation of vascular tone by DiHETEs has not been fully evaluated [36], but DiHETEs are products of epoxide hydrolase action on EETs and may produce vasodilatory effects in the renal, cerebral, and coronary arteries [36]. The conversion of EETs to DiHETEs is believed to limit the former’s effects on the vasculature [43]. For this reason, one group has probed the use of epoxide hydrolase inhibitors as a possible therapeutic target for blood pressure control [44]. However, this trend is not observed in coronary arteries, where the two metabolites have been shown to cause an equal degree of vasodilation [45]. This may be important, as cardiovascular disease is linked to cognitive decline in the elderly [46]. Increased levels of two DiHETE species that were observed in AD individuals with T2D may be indicative of increased conversion of EETs to DiHETEs, which could reduce vasodilatory effects, but this needs to be explored further. However, prior work also indicates that there is some crossover between the COX-2 and 5-LOX pathways in the synthesis of DiHETE species in activated leukocytes [47]. This is relevant in our T2D cohort because leukocyte activation occurs in acute and chronic hyperglycemia, and in T2D populations, activation of leukocytes is also associated with vascular dysfunction [48]. Thus, the increased DiHETE levels we observed could also be due to the upregulation of COX-2 independent pathways, such as 5-LOX. Other oxylipins, including 17-HDoHE and total fatty acid alcohols, have roles in inflammatory processes and PPARγ activation [27,49,50]. 17-HDoHE exhibits anti-inflammatory properties, which is of note in light of accumulating research that posits a role for inflammation in AD etiology, [51] and suggests a compensatory upregulation of 17-HDoHE in this group. Various fatty acid alcohols have also been implicated as platelet aggregation antagonists [52] and inhibitors of thromboxane A2 action [53]. The relevance of these roles to our disease population is unknown.

We observed mediocre to poor predictive performance in PLS-DA models of AD status in T2D participants and in non-diabetic participants, respectively, when including the entire serum oxylipin repertoire. This suggests that AD may elicit only a small alteration in oxylipin metabolism, and this may manifest more clearly in the T2D condition. We previously noted in this cohort that the T2D signature of non-oxylipin serum metabolites was obscured in individuals with AD [14]. These investigations point toward a subtle interactive effect between AD and T2D that differentially modulates certain metabolic pathways; however, the mechanisms that drive these effects remain to be elaborated. Despite limitations of broader blood oxylipin metabolite signatures in predicting the AD condition, select oxylipins appeared to be associated with AD and warrant further study.

Key strengths of our study were the assessment of biospecimens from individuals who had been comprehensively and robustly characterized by clinicians who specialize in AD, and the use of a targeted metabolomics platform. However, there are several limitations to note. The relatively small sample size in each group may have limited our ability to detect differences after correcting for multiple comparisons, or to identify robust discriminating metabolite signatures of AD using PLS-DA. Additionally, we used PLS-DA to assess global alterations in the serum oxylipin signature due to its ability to assess classification paradigms with a small sample size to variable ratios and also due to its robustness to multicollinearity. We recognize that PLS-DA is prone to overfitting and can lead to spurious results if not properly validated. Our cross-validation accuracy rate was <63% when including the entire serum oxylipin repertoire in either nondiabetic or diabetic. While there are no established accuracy cutoffs to define overfitting, we interpret these outcomes as poor-to-mediocre predictive performance and may suggest overfitting in the PLS-DA models. Still, we also utilized bootstrapping to identify metabolites that consistently contributed to the overall variance explained by the PLS-DA models (i.e., discrimination of AD) and found agreement with univariate results. Together, this suggests that AD does not impact the entire oxylipin metabolome, but provides evidence that AD elicits alterations in fatty acid diol metabolism. Finally, the current analyses cannot identify the tissue source(s) of the oxylipin shifts. Work to characterize tissue-specific or cerebrospinal fluid oxylipin profiles, as well as fatty acid and macronutrient metabolism in AD using isotope tracers and other methods, is warranted.

Despite these limitations, we present here the first study to assess oxylipin changes in cognitively healthy and AD subjects with and without T2D, an important emerging AD risk factor. We have identified a handful of specific oxylipins that should be explored in future studies of AD to better understand how this condition impacts synthesis and turnover, and to determine if these oxylipins regulate systems implicated in AD pathophysiology.

## 4. Materials and Methods

This study was performed using deidentified biospecimens that were obtained through the University of Kansas Alzheimer’s Disease Center (KU ADC). Specimens were collected via study #11132 that was approved by the University of Kansas Medical Center Institutional Review Board. All study participants provided informed consent, and procedures were performed in accordance with the Declaration of Helsinki of 1975, revised in 2013.

### 4.1. Metabolic and Cognitive Characterization

As described previously [14], 126 individuals (84 nondiabetic and 42 T2D individuals) participated in this study to investigate the effect of AD and T2D on the serum oxylipin profile. Of the nondiabetic cohort, 39 cognitively healthy elderly and 45 AD subjects were assessed. The T2D cohort was comprised of 23 cognitively healthy elderly and 19 AD subjects. All individuals were clinically evaluated before stratification. Participants received a Clinical Dementia Rating (CDR) and cognitive testing (UDS 2.0), with the clinical diagnosis confirmed via consensus diagnosis conference. All cognitively healthy subjects had a CDR of 0 while individuals with AD had a CDR of 0.5 or higher, with a diagnosis of either mild cognitive impairment or dementia with Alzheimer’s disease at the primary etiology. Classification of individuals as T2D was based upon World Health Organization and American Diabetes Association criteria for fasting glucose (≥126 mg/dL) or current clinical diagnosis of T2D. All T2D subjects had clinical T2D diagnosis except for 2 previously-undiagnosed individuals, who met fasting glucose criteria. Nondiabetic subjects had no current T2D diagnosis or past history of T2D, and a fasting glucose within the normal range.

Participants reported to the KU Clinical and Translational Science Unit following an overnight fast. Vital signs and anthropometric measures were documented, and body composition was assessed using dual-energy X-ray absorptiometry (DXA, Lunar Prodigy, v 11.2068). Blood samples were collected by venipuncture (typically, antecubital vein) and processed for serum before storage in multiple aliquots to minimize freeze thaws.

### 4.2. Measurement of Serum Non-Esterified Oxylipins

Metabolomics analyses were conducted by the West Coast Metabolomics Center (WCMC) at the University of California, Davis. Serum samples were shipped on dry ice from KUMC to WCMC. In-depth details the protocols have been previously published [54]. Serum samples were thawed, and 50 µL aliquots were added to Waters Ostro Sample Preparation Plate wells. Wells were then spiked with a 5 µL anti-oxidation solution and 5 µL analytical deuterated surrogates. Acetonitrile (150 µL) with 1% formic acid was forcefully added to all samples, then all samples were eluted with vacuum, dried, and reconstituted with internal standards and 1-phenyl 3-hexadecanoic acid urea at 100 nM. Samples were filtered (0.1 µm) before analysis. Extracts were separated on a Waters Acquity UPLC and detected by negative mode electrospray ionization using multiple reaction monitoring on an API 400 QTrap (AB Sciex, Framingham, MA, USA) [9,55]. Quantification of analytes assessed by internal standard methods and 5 to 7 point calibration curves (r^2^ ≥ 0.997). Data were processed with AB Sciex MultiQuant version 3.0. Final data are available in Supplemental Data.

### 4.3. Statistical Analyses

All data pre-processing and statistical analyses were conducted in the R Statistical Language (version 3.5.1). Statistical significance was considered at α ≤ 0.05, unless otherwise noted. Summed totals of oxylipin sub-species (e.g., prostanoids, fatty acid diols, etc.) were summed together and included in all analyses. R coding is available in Supplementary Materials.

#### 4.3.1. Pre-Processing of Data.

A total of 10 metabolites were removed from the analyses due to the fact that there were >50% missing data across all subjects. Metabolite data were then screened for outliers using an iterative Grubbs’ test for outliers at α ≤ 0.01. Thirteen data point outliers were detected and removed, affecting <1% of all data. Of these data points, 8 outliers were attributed to a single subject. This subject was removed after visually confirming an overall higher distribution of metabolites compared to other subjects. Missing data were imputed using the K-nearest neighbor algorithm from the Bioconductor impute package. Imputations represented 4.9% of the oxylipin data used in multivariate analyses.

#### 4.3.2. Univariate and Multivariate Assessments.

Group differences in individual metabolites were assessed by Mann–Whitney U or Kruskal–Wallis tests, depending on the number of comparative groups. *p*-values obtained from non-parametric tests were adjusted for multiple comparisons using the false discovery rate (FDR) procedure of Benjamini and Hochberg [56]. As several of the oxylipins were derived from a single fatty acid source, these data tend to contain strong multicollinearity. As this may over-penalize the univariate results, we utilized partial least squares-discriminant analysis (PLS-DA) to identify discriminant metabolites in a supervised modeling exercise. The optimal number of PLS-DA latent variables were identified by the greatest average accuracy in 10-fold cross-validation repeated 10 times. Oxylipins contributing to group discrimination in PLS-DA models were identified by variable importance projection (VIP) calculations >1 [57]. We further used bootstrapping to calculate 95% confidence intervals for VIP calculations. VIP-selected metabolites were then used to fit PLS-DA models to identify if featured metabolites could improve cross-validation results. Visualization of classifier discrimination was shown in PLS-DA scores plots. R packages used in PLS-DA modeling and feature selection included pls [58], caret [59], and boot [60,61].

## Figures and Tables

**Table 1:** Serum oxylipin concentrations and Alzheimer’s disease (AD) status in overnight-fasted nondiabetic elderly participants.

Metabolites ^1^	Cognitively Healthy (*n* = 39) ^2^	Alzheimer’s Disease (*n* = 39) ^2^	*P ^3^*	*FDR ^4^*	Sub-Class	Fatty Acid Precursor
Total Fatty Acid Alcohol	209 (110, 490)	267 (140, 500)	0.295	0.965	Fatty Acid Alcohol	-
14-HDoHE	22.9 (8.3, 52)	25.9 (14, 53)	0.376	0.965	Fatty Acid Alcohol	C22:6n3
12S-HETE	93.8 (39, 220)	119 (46, 250)	0.386	0.965	Fatty Acid Alcohol	C20:4n6
9-HOTE	1.21 (0.82, 2.1)	1.13 (0.81, 1.7)	0.470	0.965	Fatty Acid Alcohol	C18:3n3
17-HDoHE	1.72 (1.1, 3.1)	1.91 (1.4, 3.1)	0.471	0.965	Fatty Acid Alcohol	C22:6n3
5S-HEPE	0.553 (0.37, 0.93)	0.563 (0.35, 0.75)	0.486	0.965	Fatty Acid Alcohol	C20:5n3
11-HETE	1.53 (0.92, 2.2)	1.41 (0.89, 2.1)	0.580	0.965	Fatty Acid Alcohol	C20:4n6
13-HOTE	1.96 (1.3, 3)	1.88 (1.4, 2.6)	0.580	0.965	Fatty Acid Alcohol	C18:3n3
9-HODE	17.8 (14, 35)	19 (15, 24)	0.643	0.965	Fatty Acid Alcohol	C18:2n6
5-HETE	3.72 (2.7, 4.8)	3.75 (3.3, 4.5)	0.668	0.965	Fatty Acid Alcohol	C20:4n6
15-HETE	3.1 (2.2, 5.4)	3.68 (2.4, 5)	0.681	0.965	Fatty Acid Alcohol	C20:4n6
12S-HEPE	5.72 (2, 12)	5 (2.3, 13)	0.695	0.965	Fatty Acid Alcohol	C20:5n3
15-HEPE	0.313 (0.21, 0.65)	0.369 (0.21, 0.56)	0.762	0.965	Fatty Acid Alcohol	C20:5n3
9-HEPE	0.235 (0.17, 0.46)	0.268 (0.16, 0.41)	0.824	0.972	Fatty Acid Alcohol	C20:5n3
8S-HETE	0.907 (0.62, 1.3)	0.876 (0.68, 1.3)	0.908	0.972	Fatty Acid Alcohol	C20:4n6
9-HETE	0.879 (0.61, 1.2)	0.864 (0.61, 1.3)	0.915	0.972	Fatty Acid Alcohol	C20:4n6
13S-HODE	26.7 (19, 46)	27.6 (20, 36)	0.957	0.972	Fatty Acid Alcohol	C18:2n6
4-HDoHE	0.596 (0.32, 1.1)	0.592 (0.4, 0.89)	0.965	0.972	Fatty Acid Alcohol	C22:6n3
**14,15-DiHETrE**	**0.633 (0.51, 0.77)**	**0.738 (0.64, 0.89)**	**0.021**	**0.510**	**Fatty Acid Diol**	**C18:3n3**
**11,12-DiHETrE**	**0.509 (0.41, 0.62)**	**0.599 (0.46, 0.75)**	**0.027**	**0.510**	**Fatty Acid Diol**	**C20:4n6**
**5,6-DiHETrE**	**0.332 (0.23, 0.43)**	**0.38 (0.3, 0.45)**	**0.044**	**0.514**	**Fatty Acid Diol**	**C20:4n6**
**8,9-DiHETrE**	**0.296 (0.23, 0.38)**	**0.367 (0.29, 0.42)**	**0.045**	**0.514**	**Fatty Acid Diol**	**C20:4n6**
LTB4	0.763 (0.49, 1.4)	1.02 (0.68, 1.6)	0.059	0.564	Fatty Acid Diol	C20:4n6
19,20-DiHDPE	1.55 (1.3, 2)	1.74 (1.3, 2.6)	0.155	0.930	Fatty Acid Diol	-
9,10-DiHODE	0.182 (0.11, 0.27)	0.26 (0.14, 0.34)	0.163	0.930	Fatty Acid Diol	C18:3n3
9,10-e-DiHO	5.02 (4.2, 6)	4.92 (4.2, 5.5)	0.437	0.965	Fatty Acid Diol	C18:0
17,18-DiHETE	4.97 (2.9, 7.2)	5.41 (3.2, 8.6)	0.448	0.965	Fatty Acid Diol	C20:5n3
6-trans-LTB4	0.209 (0.11, 0.29)	0.214 (0.14, 0.35)	0.596	0.965	Fatty Acid Diol	C20:4n6
15,16-DiHODE	6.52 (5.2, 9.6)	7.43 (5.5, 11)	0.646	0.965	Fatty Acid Diol	C18:3n3
9,10-DiHOME	3.08 (2.5, 4.2)	3.2 (2.5, 4.4)	0.701	0.965	Fatty Acid Diol	C18:2n6
Total Fatty Acid Diol	30 (27, 32)	28.3 (24, 36)	0.872	0.972	Fatty Acid Diol	-
14,15-DiHETE	0.605 (0.35, 0.92)	0.548 (0.41, 0.81)	0.931	0.972	Fatty Acid Diol	C20:5n3
12(13)Ep-9-KODE	5.35 (2, 11)	4.22 (1.6, 9.4)	0.471	0.965	Fatty Acid Epoxide	C18:3n3
12(13)-EpOME	2.04 (1.5, 3.1)	2.38 (1.6, 3.2)	0.580	0.965	Fatty Acid Epoxide	C18:2n6
Total Fatty Acid Epoxide	15.4 (9.9, 23)	12.5 (8.9, 23)	0.580	0.965	Fatty Acid Epoxide	-
9,10-EpO	1.35 (1.1, 1.7)	1.31 (1.1, 1.6)	0.621	0.965	Fatty Acid Epoxide	C18:0
19(20)-EpDPE	0.179 (0.14, 0.24)	0.197 (0.14, 0.29)	0.794	0.972	Fatty Acid Epoxide	C22:6n3
9(10)-EpOME	0.829 (0.57, 1.2)	0.924 (0.65, 1.4)	0.858	0.972	Fatty Acid Epoxide	C18:2n6
15,16-EpODE	1.33 (0.86, 1.9)	1.39 (0.87, 2.2)	0.922	0.972	Fatty Acid Epoxide	C18:3n3
Total Fatty Acid Ketone	24.6 (20, 54)	22.8 (14, 31)	0.150	0.930	Fatty Acid Ketone	-
9-KODE	14.5 (9.6, 28)	13 (6.9, 20)	0.168	0.930	Fatty Acid Ketone	C18:2n6
15-KETE	0.287 (0.18, 0.48)	0.221 (0.15, 0.37)	0.179	0.930	Fatty Acid Ketone	C20:4n6
13-KODE	5.87 (3.3, 12)	4.26 (2.2, 8.5)	0.252	0.965	Fatty Acid Ketone	C18:2n6
5-KETE	0.342 (0.25, 0.55)	0.36 (0.24, 0.45)	0.574	0.965	Fatty Acid Ketone	C20:4n6
9,12,13-TriHOME	3.68 (2.7, 4.3)	3.54 (2.3, 4.7)	0.735	0.965	Fatty Acid Triol	C18:2n6
**10-Nitrooleate**	**1.46 (1.1, 2.3)**	**1.2 (0.88, 1.6)**	**0.026**	**0.510**	**Nitro-fatty Acid**	**C18:1n9**
6-keto-PGF1alpha	0.22 (0.2, 0.24)	0.213 (0.19, 0.23)	0.315	0.965	Prostanoid	C20:4n6
PGF2alpha	0.422 (0.25, 0.71)	0.518 (0.29, 0.88)	0.507	0.965	Prostanoid	C20:4n6
Total Prostanoid	0.745 (0.62, 1.1)	0.816 (0.6, 1.2)	0.714	0.965	Prostanoid	-
15-deoxy PGJ2	0.115 (0.11, 0.12)	0.114 (0.11, 0.12)	0.727	0.965	Prostanoid	C20:4n6
TXB2	3.24 (0.16, 20)	0.839 (0.2, 14)	0.534	0.965	Thromboid	C20:4n6
Total C18:0 species	6.49 (5.3, 7.1)	6.14 (5.6, 6.9)	0.456	0.965	-	C18:0
Total C18:2n6 species	73.6 (58, 140)	76.8 (56, 100)	0.574	0.965	-	C18:2n6
Total C22:6n3 species	45.3 (26, 98)	37.8 (19, 83)	0.723	0.965	-	C22:6n3
Total C20:5n3 species	14.3 (8.9, 26)	13.9 (9, 20)	0.747	0.965	-	C20:5n3
Total C20:4n6 species	184 (78, 390)	138 (96, 290)	0.888	0.972	-	C20:4n6
Total C18:3n3 species	20.5 (13, 31)	19.3 (14, 28)	0.972	0.972	-	C18:3n3

^1^ Assessed by UPLC-MS/MS. Units are nmol/mL (µM); ^2^ Values are median (1st quartile, 3rd quartile); ^3^ Derived from the Mann–Whitney U test; ^4^ Derived from Benjamini and Hochberg False Discovery Rate (FDR) correction; Bold analytes are significantly different between groups before FDR correction.

**Table 2:** Serum oxylipin concentrations and AD status in overnight-fasted Type 2 Diabetic elderly participants.

Metabolites ^1^	Cognitive Healthy (*n* = 22) ^2^	Alzheimer’s Disease (*n* = 19) ^2^	*P ^3^*	*FDR ^4^*	Sub-Class	Fatty Acid Precursor
**17-HDoHE**	**1.57 (1.1, 2.2)**	**3.22 (2.1, 4.5)**	**0.014**	**0.523**	**Fatty Acid Alcohol**	**C22:6n3**
**Total Fatty Acid Alcohol**	**228 (120, 290)**	**421 (250, 690)**	**0.041**	**0.523**	**Fatty Acid Alcohol**	**-**
12S-HEPE	4.06 (3.4, 8)	6.94 (3.5, 27)	0.156	0.697	Fatty Acid Alcohol	C20:5n3
9-HODE	20.1 (17, 26)	18.2 (15, 24)	0.237	0.697	Fatty Acid Alcohol	C18:2n6
12S-HETE	125 (49, 190)	146 (86, 320)	0.259	0.697	Fatty Acid Alcohol	C20:4n6
15-HEPE	0.242 (0.19, 0.55)	0.357 (0.21, 0.83)	0.270	0.697	Fatty Acid Alcohol	C20:5n3
15-HETE	3.67 (2.6, 4.4)	3.78 (2.7, 6.5)	0.281	0.697	Fatty Acid Alcohol	C20:4n6
9-HEPE	0.206 (0.14, 0.37)	0.275 (0.14, 0.47)	0.318	0.697	Fatty Acid Alcohol	C20:5n3
14-HDoHE	27.5 (16, 42)	37.5 (19, 110)	0.344	0.697	Fatty Acid Alcohol	C22:6n3
9-HOTE	1.2 (0.94, 1.6)	1.06 (0.71, 1.6)	0.445	0.724	Fatty Acid Alcohol	C18:3n3
5S-HEPE	0.473 (0.33, 0.65)	0.553 (0.38, 0.85)	0.460	0.729	Fatty Acid Alcohol	C20:5n3
13S-HODE	26.3 (22, 37)	25.8 (22, 42)	0.727	0.958	Fatty Acid Alcohol	C18:2n6
4-HDoHE	0.577 (0.44, 0.76)	0.491 (0.37, 1.1)	0.791	0.958	Fatty Acid Alcohol	C22:6n3
5-HETE	3.75 (3, 4.9)	3.69 (3, 4.9)	0.846	0.958	Fatty Acid Alcohol	C20:4n6
8S-HETE	0.832 (0.69, 1)	0.85 (0.71, 1.1)	0.846	0.958	Fatty Acid Alcohol	C20:4n6
13-HOTE	1.51 (1.3, 2.2)	1.64 (1.2, 2.8)	0.867	0.958	Fatty Acid Alcohol	C18:3n3
11-HETE	1.17 (1, 1.8)	1.35 (0.92, 1.8)	0.907	0.958	Fatty Acid Alcohol	C20:4n6
9-HETE	0.794 (0.69, 0.99)	0.789 (0.69, 0.96)	0.969	0.986	Fatty Acid Alcohol	C20:4n6
**17,18-DiHETE**	**3.86 (2.9, 4.1)**	**4.98 (4.2, 8)**	**0.024**	**0.523**	**Fatty Acid Diol**	**C20:5n3**
**14,15-DiHETE**	**0.42 (0.36, 0.6)**	**0.7 (0.61, 0.81)**	**0.044**	**0.523**	**Fatty Acid Diol**	**C20:5n3**
9,10-DiHODE	0.208 (0.084, 0.29)	0.341 (0.18, 0.59)	0.062	0.523	Fatty Acid Diol	C18:3n3
15,16-DiHODE	7.26 (6.1, 10)	5.19 (4, 8.1)	0.065	0.523	Fatty Acid Diol	C18:3n3
6-trans-LTB4	0.211 (0.14, 0.27)	0.318 (0.17, 0.41)	0.134	0.697	Fatty Acid Diol	C20:4n6
19,20-DiHDPE	1.46 (1.4, 1.8)	2.02 (1.4, 2.3)	0.198	0.697	Fatty Acid Diol	-
5,6-DiHETrE	0.39 (0.26, 0.42)	0.401 (0.31, 0.52)	0.293	0.697	Fatty Acid Diol	C20:4n6
9,10-e-DiHO	5.04 (4.5, 5.7)	5.45 (4.3, 9.2)	0.305	0.697	Fatty Acid Diol	C18:0
8,9-DiHETrE	0.341 (0.27, 0.39)	0.38 (0.29, 0.46)	0.357	0.697	Fatty Acid Diol	C20:4n6
Total Fatty Acid Diol	27.4 (22, 31)	31.3 (26, 33)	0.426	0.724	Fatty Acid Diol	-
14,15-DiHETrE	0.692 (0.53, 0.83)	0.71 (0.56, 0.93)	0.578	0.876	Fatty Acid Diol	C18:3n3
9,10-DiHOME	2.71 (2.3, 3.9)	2.98 (2.1, 4.2)	0.697	0.958	Fatty Acid Diol	C18:2n6
11,12-DiHETrE	0.567 (0.43, 0.7)	0.548 (0.44, 0.82)	0.867	0.958	Fatty Acid Diol	C20:4n6
LTB4	0.758 (0.43, 1.1)	0.783 (0.3, 1.5)	0.990	0.990	Fatty Acid Diol	C20:4n6
15,16-EpODE	1.56 (1.2, 2.3)	0.887 (0.69, 1.9)	0.073	0.523	Fatty Acid Epoxide	C18:3n3
9,10-EpO	1.55 (1.2, 2)	1.19 (1, 1.7)	0.164	0.697	Fatty Acid Epoxide	C18:0
12(13)Ep-9-KODE	2.08 (0.87, 6.7)	3.38 (2, 10)	0.232	0.697	Fatty Acid Epoxide	C18:3n3
Total Fatty Acid Epoxide	15.1 (8.6, 18)	18.4 (15, 21)	0.379	0.697	Fatty Acid Epoxide	-
19(20)-EpDPE	0.205 (0.15, 0.24)	0.218 (0.15, 0.29)	0.600	0.876	Fatty Acid Epoxide	C22:6n3
12(13)-EpOME	1.85 (1.2, 3.1)	2.07 (1.2, 3)	0.826	0.958	Fatty Acid Epoxide	C18:2n6
9(10)-EpOME	0.778 (0.61, 1.4)	0.781 (0.68, 1.2)	0.887	0.958	Fatty Acid Epoxide	C18:2n6
9-KODE	12.6 (11, 20)	10.2 (7.4, 15)	0.141	0.697	Fatty Acid Ketone	C18:2n6
13-KODE	4.79 (3.9, 7.5)	4.04 (2.3, 7.3)	0.371	0.697	Fatty Acid Ketone	C18:2n6
5-KETE	0.287 (0.24, 0.54)	0.284 (0.23, 0.34)	0.688	0.958	Fatty Acid Ketone	C20:4n6
15-KETE	0.218 (0.15, 0.42)	0.198 (0.17, 0.26)	0.905	0.958	Fatty Acid Ketone	C20:4n6
Total Fatty Acid Ketone	19.9 (18, 35)	21.2 (17, 34)	0.905	0.958	Fatty Acid Ketone	-
9,12,13-TriHOME	3.8 (2.3, 5.4)	3.25 (2.2, 4.1)	0.259	0.697	Fatty Acid Triol	C18:2n6
10-Nitrooleate	1.23 (0.86, 2.2)	1.09 (0.75, 1.4)	0.254	0.697	Nitro-fatty Acid	C18:1n9
6-keto-PGF1alpha	0.213 (0.19, 0.23)	0.223 (0.21, 0.24)	0.189	0.697	Prostanoid	C20:4n6
Total Prostanoid	0.71 (0.58, 0.88)	0.953 (0.68, 1.1)	0.314	0.697	Prostanoid	-
15-deoxy PGJ2	0.115 (0.11, 0.12)	0.121 (0.11, 0.13)	0.337	0.697	Prostanoid	C20:4n6
PGF2alpha	0.375 (0.26, 0.53)	0.494 (0.35, 0.75)	0.404	0.719	Prostanoid	C20:4n6
TXB2	0.779 (0.49, 9.9)	0.876 (0.25, 5.8)	0.940	0.974	Thromboid	C20:4n6
Total C22:6n3 species	19.2 (11, 40)	118 (75, 140)	0.073	0.523	-	C22:6n3
Total C18:2n6 species	76 (64, 93)	64.7 (57, 90)	0.190	0.697	-	C18:2n6
Total C20:5n3 species	10 (7.7, 23)	14.9 (7.9, 27)	0.440	0.724	-	C20:5n3
Total C18:0 species	6.2 (5.7, 7.4)	6.77 (5.7, 11)	0.596	0.876	-	C18:0
Total C20:4n6 species	167 (130, 210)	182 (130, 330)	0.734	0.958	-	C20:4n6
Total C18:3n3 species	19.6 (16, 24)	19.2 (13, 24)	0.778	0.958	-	C18:3n3

^1^ Assessed by UPLC-MS/MS. Units are nmol/mL (µM); ^2^ Values are median (1st quartile, 3rd quartile); ^3^ Derived from the Mann–Whitney U test; ^4^ Derived from Benjamini and Hochberg FDR correction; Analytes in bold are significantly different between groups before FDR correction.

**Table 3:** Directionality of effects within metabolic groups as identified via nonparametric analyses.

	Nondiabetic Group (*n* = 84)	T2D Group (*n* = 42)	Notable Roles
10-nitrooleate	Lower in AD	-	Nitric oxide synthase regulation [15] Inhibition of neutrophil chemotaxis via PPARγ activation [16,17] Damage mediation after reperfusion in cardiac ischemia [18]
14,15-DiHETrE	Higher in AD	-	Promotes vasodilation in preclinical models [19,20] Elevated in pregnancy-related hypertension [21]
11,12-DiHETrE	Higher in AD	-	Increased odds of vascular events* [22] Potential marker of ventricular arrhythmia [23] Promotes vasodilation in preclinical models [19,20]
8,9-DiHETrE	Higher in AD	-	Increased odds of vascular events* [22] Potential marker of ventricular arrhythmia [23] Promotes vasodilation in preclinical models [19] Elevated after treatment of ibuprofen in humans [24]
5,6-DiHETrE	Higher in AD	-	Potential marker of ventricular arrhythmia [23] Vasodilation in mouse due to increased nitric oxide availability [19] Elevated after treatment of ibuprofen in healthy males [24] Promotes vasodilation in preclinical models [20]
14,15-DiHETE	-	Higher in AD	Inhibition of platelet aggregation [25]
17,18-DiHETE	-	Higher in AD	Potential marker of ventricular arrhythmia [23] Inhibition of platelet aggregation [25]
17-HDoHE	-	Higher in AD	Anti-inflammatory action in preclinical models [26] PPARγ agonist in cell models [27]

Notable roles of oxylipins identified as being different between AD and cognitively healthy elderly individuals. Overall, the identified oxylipin species are consistently linked to vascular or inflammatory outcomes in prior studies. *Vascular events are defined as the occurrence of a transient ischemic attack, cerebrovascular accidents, stable angina, and acute coronary syndrome.

## Supplementary Materials

The following are available online at https://www.mdpi.com/2218-1989/9/9/177/s1: Final data, R coding, oxylipin class key.
